# Enhanced base editing by co-expression of free uracil DNA glycosylase inhibitor

**DOI:** 10.1038/cr.2017.111

**Published:** 2017-08-29

**Authors:** Lijie Wang, Wei Xue, Lei Yan, Xiaosa Li, Jia Wei, Miaomiao Chen, Jing Wu, Bei Yang, Li Yang, Jia Chen

**Affiliations:** 1 School of Life Science and Technology, ShanghaiTech University, Shanghai 201210, China; 2 Shanghai Institute of Biochemistry and Cell Biology, Chinese Academy of Sciences, Shanghai 200031, China; 3 University of Chinese Academy of Sciences, Beijing 100049, China; 4 Key Laboratory of Computational Biology, CAS-MPG Partner Institute for Computational Biology, Shanghai Institutes for Biological Sciences, Chinese Academy of Sciences, Shanghai 200031, China; 5 Shanghai Institute for Advanced Immunochemical Studies, ShanghaiTech University, Shanghai 201210, China

## Dear Editor,

Base editors (BEs) have been recently developed by combining the APOBEC (apolipoprotein B mRNA editing enzyme, catalytic polypeptide-like)/AID (activation-induced deaminase) cytidine deaminase family members^1^ with the CRISPR/Cas9 system to perform targeted C-to-T base editing^2, 3, 4, 5,6,7,8^. Mechanistically, Cas9 variant-fused APOBEC/AID is directed to target site by sgRNA, introducing C-to-T substitution at the single-base level^2,3,4^. Compared to earlier generations of BEs (BE1 and BE2), the latest BE3 achieved much higher base editing frequencies by substituting catalytically-dead Cas9 (dCas9) with Cas9 nickase (nCas9)^2^. Because BEs achieve gene corrections without introducing DNA double-strand breaks (DSBs), unwanted indels converted from DSBs through non-homologous end joining (NHEJ) were thought to be excluded in base editing. However, non-negligible levels of indels (∼4%-12% in published cases^2,3^) were still observed in BE3-mediated base editing. In addition, unwanted non-C-to-T (i.e., C-to-A or C-to-G) substitutions were observed, and the frequencies of C-to-A/C-to-G substitutions could be as high as that of C-to-T substitution in some examined cases^5^. The existence of unwanted indels and C-to-A/C-to-G substitutions compromises the fidelity of base editing outcome.

Thus, understanding what causes the formation of those unwanted indels and C-to-A/C-to-G substitutions during base editing will help achieve a cleaner yield of BE3. Ideally, along with the U:G mismatch introduced by APOBEC-mediated cytidine deamination on the non-target strand (NTS), the nCas9-generated nick on the sgRNA target strand (TS) activates mismatch repair (MMR) pathway^9,10^ to excise the nicked TS (Supplementary information, Figure S1A). Subsequent TS DNA re-synthesis using the edited NTS as a template converts the original U:G mismatch into a U:A pair, whereby the desired C-to-T substitution is achieved after DNA replication (Supplementary information, Figure S1B). However, the U on the single-stranded NTS could also be transformed into an apurinic/apyrimidinic (AP) site by various DNA glycosylases, including uracil DNA glycosylase (UDG)^11^ (Supplementary information, Figure S1C), to trigger other DNA repair pathways. For instance, AP endonuclease-mediated cleavage or spontaneous breakage of AP site-containing ssDNA could trigger NHEJ to form indels (Supplementary information, Figure S1C, left); additionally, translesion synthesis (TLS) over the AP site by TLS DNA polymerase could result in a C-to-A or C-to-G substitution (Supplementary information, Figure S1C, right). Thus, it is tempting to speculate that preventing the transformation of the APOBEC-generated U into AP site on the single-stranded NTS could reduce unwanted indels and non-C-to-T substitutions. Uracil DNA glycosylase inhibitor (UGI) domain was fused to nCas9 in BE3 to prevent the transformation of U into AP site. To test the importance of UGI in base editing, we first removed the fused UGI in BE3. Consistent with our hypothesis mentioned above (Supplementary information, Figure S1C), the UGI-deleted BE3 (BE3-ΔUGI; Supplementary information, Figure S2A) was less competent in base editing (Supplementary information, Figure S2B-S2L). Compared to BE3, BE3-ΔUGI induced higher unwanted indel frequencies and lower desired C-to-T editing (Supplementary information, Figure S2B-S2D, *P* < 0.01 and Figure S2E-S2G, *P* < 10^−5^). As a consequence, the ratios of C-to-T editing to indels decreased considerably (Supplementary information, Figure S2H-S2J, *P* < 10^−6^). Meanwhile, the unwanted C-to-A/C-to-G substitutions also increased in the absence of UGI (Supplementary information, Table S2), leading to a significant reduction of C-to-T over C-to-A/C-to-G substitutions (Supplementary information, Figure S2K-S2L, *P* < 10^−4^). These results thus indicated that preventing the transformation of U into AP site is pivotal for efficient and high-fidelity base editing.

**Figure 1:**
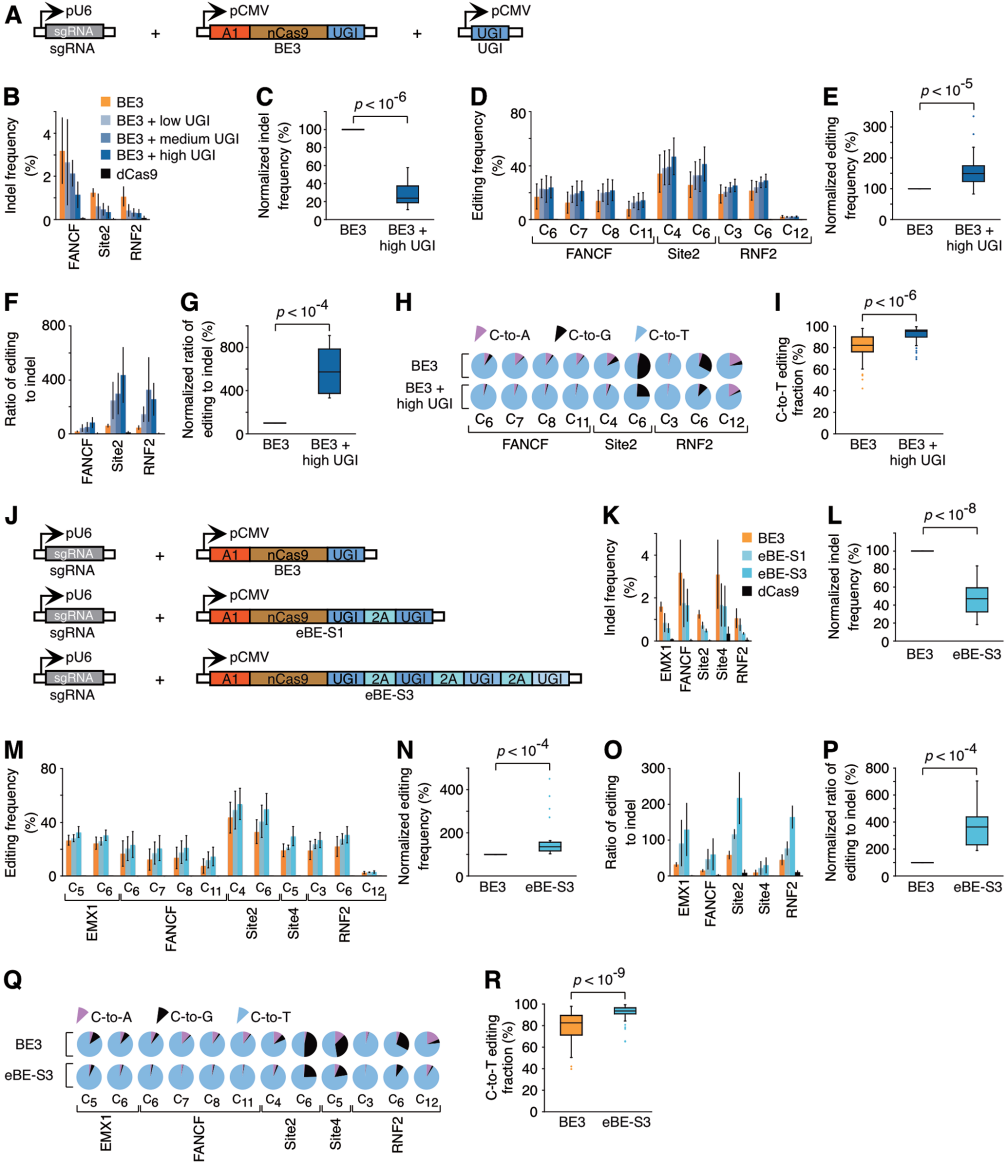
Enhanced base editing system. **(A-I)** Enhanced base editing by co-expressing BE3 and free UGI from separate vectors in 293FT cells. Schematic diagram illustrating the design of sgRNA, BE3 and UGI expression vectors **(A)**. The indel frequency **(B)**, the C-to-T editing frequency at the indicated position of the sgRNA target region **(D)**, the ratio of desired C-to-T editing to unwanted indels **(F)** and the fractions of C-to-T, C-to-A and C-to-G substitutions **(H)** were individually determined at the specified genomic sites for the indicated conditions and plotted as follows: orange represents BE3, faint blue represents BE + low UGI, blue represents BE3 + medium UGI, dark blue represents BE3 + high UGI and black represents dCas9. The positions of edited Cs in the sgFANCF, sgSite2 and sgRNF2 target regions were indicated with the base distal from the PAM set as position 1. Statistical analyses highlighted the significant differences between BE3 (orange) and BE3 + high UGI (dark blue) in indel frequency **(C)**, in C-to-T editing frequency at the indicated position within sgRNA target region **(E)**, in the ratio of desired C-to-T editing to unwanted indels **(G)** and in the fraction of C-to-T substitution **(I)**. **(J-R)** Enhanced base editing by eBE-S1 and eBE-S3 in 293FT cells. Schematic diagram illustrating the design of sgRNA, BE3, eBE-S1 and eBE-S3 expression vectors **(J)**. The indel frequency **(K)**, the C-to-T editing frequency **(M)**, the ratio of desired C-to-T editing to unwanted indels **(O)** and the fractions of C-to-T, C-to-A and C-to-G substitutions **(Q)** were individually determined at the indicated genomic sites for BE3 (orange), eBE-S1(faint cyan) and eBE-S3 (cyan). The positions of edited Cs in the sgEMX1, sgFANCF, sgSite2, sgSite4 and sgRNF2 target regions were indicated with the base distal from the PAM set as position 1. Statistical analyses highlighted the significant differences between BE3 (orange) and eBE-S3 (cyan) in indel frequency **(L)**, in the C-to-T editing frequency **(N)**, in the ratio of desired C-to-T editing to unwanted indels **(P)** and in the fraction of C-to-T substitution **(R)**. **(B**, **D**, **F**, **K**, **M** and **O)** Error bars (±), standard deviations of 3 replicates. **(C**, **E**, **G**, **I**, **L**, **N**, **P** and **R)** *P* values, one-tailed Student's *t*-test.

Although UGI was fused to nCas9 in BE3, indels were still observed in reported studies^2,3^. Such a phenomenon suggests that additional UGI activity may be required to further improve the efficiency and fidelity of BE3-mediated base editing. We therefore tested this hypothesis by co-expressing UGI *in trans* with BE3. After co-transfection of UGI *in trans* with sgRNA/BE3 in 293FT cells (Figure 1A and Supplementary information, Figure S3A and S3B), we applied deep-sequencing to determine the indel and base substitution frequencies at three sgRNA target sites. Compared to BE3 alone, co-expressing BE3 and UGI *in trans* evidently reduced the indel frequencies (Figure 1B and 1C, *P* < 10^−6^, Supplementary information, Table S1) and promoted C-to-T editing frequencies at target bases (Figure 1D and 1E, *P* < 10^−5^; Supplementary information, Table S2). Specifically, the expression level of UGI is positively correlated with the ratio of C-to-T editing to indels (Figure 1F). When a high level of free UGI is present, the ratio of desired base editing to unwanted indels increased by ∼6-fold (Figure 1G, *P* < 10^−4^). At the same time, the unwanted C-to-A/C-to-G substitutions were also suppressed in most tested cases by free UGI expression (Supplementary information, Table S2), resulting in a significant increase of C-to-T over C-to-A/C-to-G substitutions (Figure 1H and 1I, *P* < 10^−6^). We noticed that the variations among biological replicates were not trivial (Figure 1B, 1D and 1F, standard deviation represented by error bar), which could be explained by the different transfection efficiencies among replicates. To exclude the influence of transfection efficiency among different biological replicates, we normalized the indel frequencies, C-to-T editing frequencies and the ratios of editing to indels induced in BE3/UGI co-expression by those induced in paired BE3 tests. As illustrated in Supplementary information, Figure S3C-S3E, consistently better base editing effects were observed in BE3/UGI co-expression than in BE3. Moreover, the statistical analysis indicates that those improving effects conferred by high level of free UGI were highly significant (Figure 1C, 1E and 1G, *P* values were all within the range of 10^−6^ to 10^−4^). These results indicated that additional free UGI could reduce AP site formation on single-stranded NTS, thereby suppressing the generation of unwanted indels and C-to-A/C-to-G substitutions and simultaneously increasing the desired C-to-T editing.

We next sought to set up the enhanced BE (eBE) more conveniently by using a single vector to co-express BE3 with either one (eBE-S1) or three (eBE-S3) copies of 2A-UGI sequence (Figure 1J). After being transfected into 293FT cells together with five sgRNAs targeting different genomic loci, both eBEs showed lower indel frequencies and higher C-to-T editing frequencies than the original BE3 (Figure 1K and 1M; Supplementary information, Tables S1 and S2); eBE-S3, with three copies of 2A-UGI and the highest level of UGI expression (Supplementary information, Figure S4A), displayed the most robust and highly significant effect (Figure 1K-1N, *P* < 10^−8^-10^−4^; Supplementary information, Figure S4B and S4C, Tables S1 and S2). Consistently, the ratios of C-to-T editing to indels were elevated when either eBE was used (Figure 1O and 1P, *P* < 10^−4^ for eBE-S3; Supplementary information, Figure S4D). Moreover, the C-to-A/C-to-G substitutions were also suppressed by eBEs (Supplementary information, Table S2) and eBE-S3 induced a highly significant increase of C-to-T fractions over C-to-A/C-to-G (Figure 1Q and 1R, *P* < 10^−9^). It is worth noting that the nCas9-fused UGI domain is still important for achieving high fidelity of base editing, even when high levels of free UGI is present (data not shown). Such facts corroborate the importance of preventing U from transforming into AP site and are consistent with our hypothesis presented above (Supplementary information, Figure S1C).

Next, we tested the effects of co-expressing BE3 and free UGI in another cell line, HeLa (Supplementary information, Figure S5). Compared to BE3, co-expressing free UGI from a separate or the same vector both induced significantly lower indel frequencies (Supplementary information, Figure S5B-S5D), higher C-to-T editing frequencies (Supplementary information, Figure S5E-S5G), higher ratios of C-to-T editing to indels (Supplementary information, Figure S5H-S5J) and higher C-to-T fractions over C-to-A/C-to-G (Supplementary information, Figure S5K and S5L). Taken together, these results indicated that our enhanced base editing system can improve the efficiency and outcome fidelity of base editing, leading to more accurate gene editing at the single-base level.

In conclusion, we have developed an enhanced base editing system by co-expressing BE3 together with free UGI. This enhanced base editing system not only suppressed the formation of unwanted indels and substitutions but also increased the frequency of C-to-T editing, thereby improving both the fidelity and efficiency of base editing. In conditions such as therapy-related applications of BEs, the 'cleanness' of editing is pursued. Our finding thus provides a method to further improve BEs for cleaner editing outcomes. Since new BEs utilizing nCas9s with altered PAMs have recently been developed^4^, our enhanced base editing strategy reported here could also be used to improve the fidelity and efficiency of these newly emerged BEs.

Materials and Methods are available in Supplementary information, Data S1, Tables S3 and S4.

## Supplementary Information

Supplementary information, Figure S1 Supplementary information, Figures 1–5

Supplementary information, Table S1 Calculation of indels.

Supplementary information, Table S2 Calculation of base substitutions.

Supplementary information, Table S3 Oligos used for plasmid construction.

Supplementary information, Table S4 sgRNA target sequences and PCR primers for amplifying sgRNA-targeted genomic regions.
