# Supplementary material for: Enhanced base editing by co-expression of free uracil DNA glycosylase inhibitor
Source: Cell Res. 2017 Aug 29;27(10):1289–92. doi: 10.1038/cr.2017.111 (PMC5630677; doi:10.1038/cr.2017.111)
Supplement: Supplementary information, Figure S1 — Supplementary information, Figures 1–5 [file cr2017111x1.pdf]

SUPPLEMENTARY INFORMATION

Supplementary Figure Legends

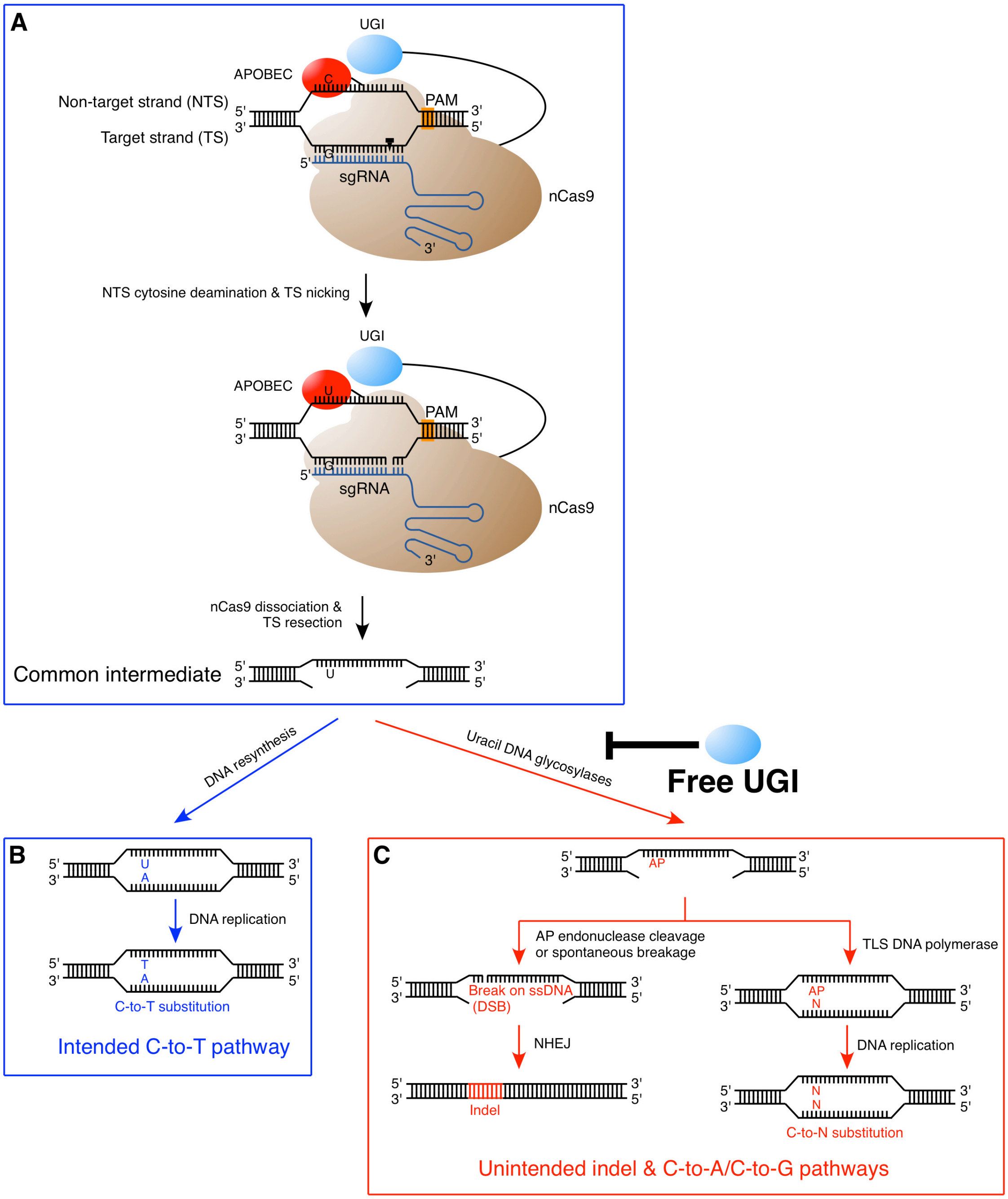

Figure S1. Proposed model for BE3-triggered indels and C-to-N base substitutions.

During the sgRNA/BE3-mediated base editing, C-to-U deamination was catalyzed by

APOBEC on the NTS, and the TS was nicked by nCas9 to trigger mismatch repair (MMR) pathway. Subsequent MMR process would resect the nicked TS and leave the NTS single-stranded (A). If the U on the NTS was copied, the subsequent DNA replication would finally lead to a C-to-T substitution (B). Alternatively, the U on the single-stranded NTS would be removed by various uracil DNA glycosylases and be converted into an AP site. The AP site could either be further converted to a DSB that triggers NHEJ to form indels (C, left) or lead to an unwanted C-to-N substitution by translesion DNA synthesis (C, right). Adding free UGI would suppress the UDG pathway, thereby increasing the frequency of C-to-T base editing and reducing the formation of unintended indels and C-to-A/C-to-G substitutions.

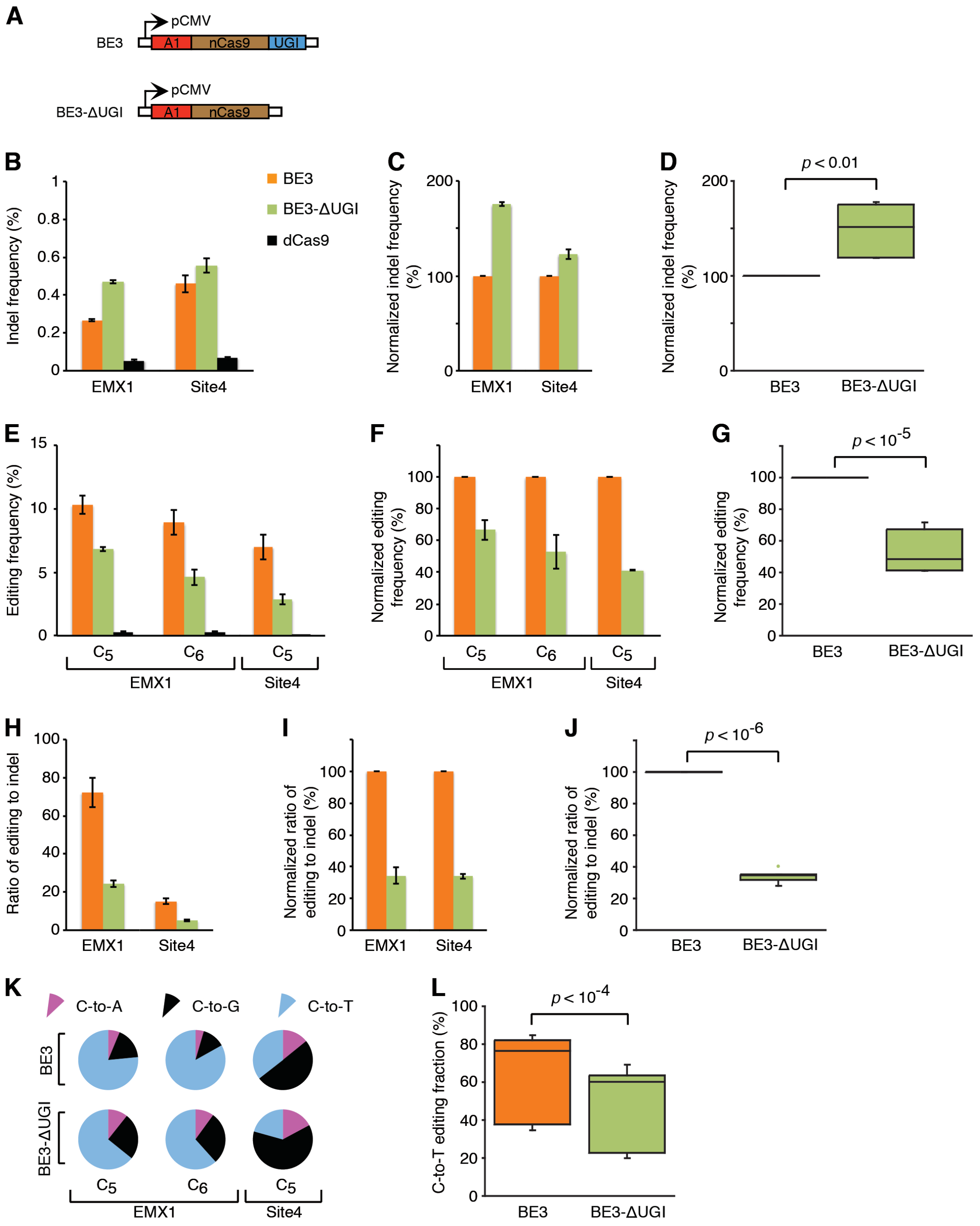

Figure S2. UGI is indispensable for BE3-mediated base editing. (A) Schematic diagram illustrating the design of expression vectors BE3 and BE3-ΔUGI. The indel frequency (B), the C-to-T editing frequency at indicated position of the sgRNA target

region (E), the ratio of desired editing to unwanted indels (H) and the fractions of C-to-T, C-to-A and C-to-G substitutions (K) were individually determined at the specified genomic sites for indicated conditions and plotted as following: orange represents BE3, light green represents BE3-ΔUGI. The normalized indel frequency (C), the normalized C-to-T editing frequency (F), the normalized ratio of desired editing to unwanted indels (I) were also shown, setting the ones induced by original BE3 as 100%. (D, G, J, L) Statistical analyses highlighted the significant differences between BE3 (orange) and BE3-ΔUGI (light green) in indel frequency (D), in C-to-T editing frequency at indicated position of sgRNA target region (G), in the ratio of desired C-to-T editing to unwanted indels (J) and in the fraction of C-to-T substitution (L).

(B, C, E, F, H and I) Error bars (±), standard deviations of 3 replicates.

(D, G, J, L) *P* values, one-tailed Student's T test.

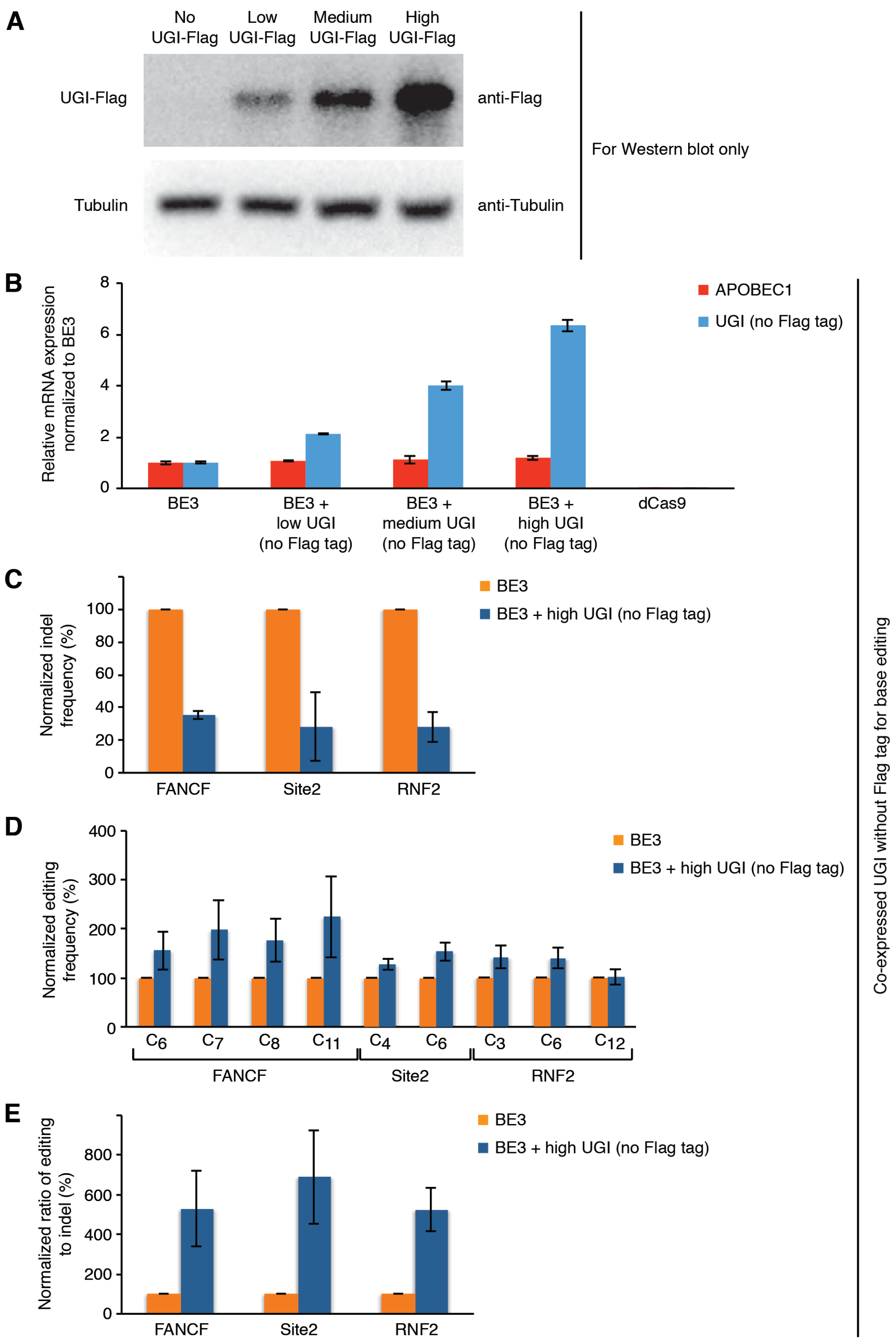

Figure S3. Enhanced base editing by co-expressing free UGI and BE3 from separate vectors. (A) The UGI-Flag fusion protein expression level was examined by Western blot. (B) The APOBEC1 and UGI mRNA expression levels were examined by RT-qPCR

respectively. (C-E) The normalized indel frequency (C), the normalized C-to-T editing frequency at indicated position (D), the normalized ratio of desired editing to unwanted indels (E) for BE3 (orange) and BE3+high UGI (dark blue) were shown, setting the ones induced by BE3 as 100%.

(B-E) Error bars (±), standard deviations of 3 replicates.

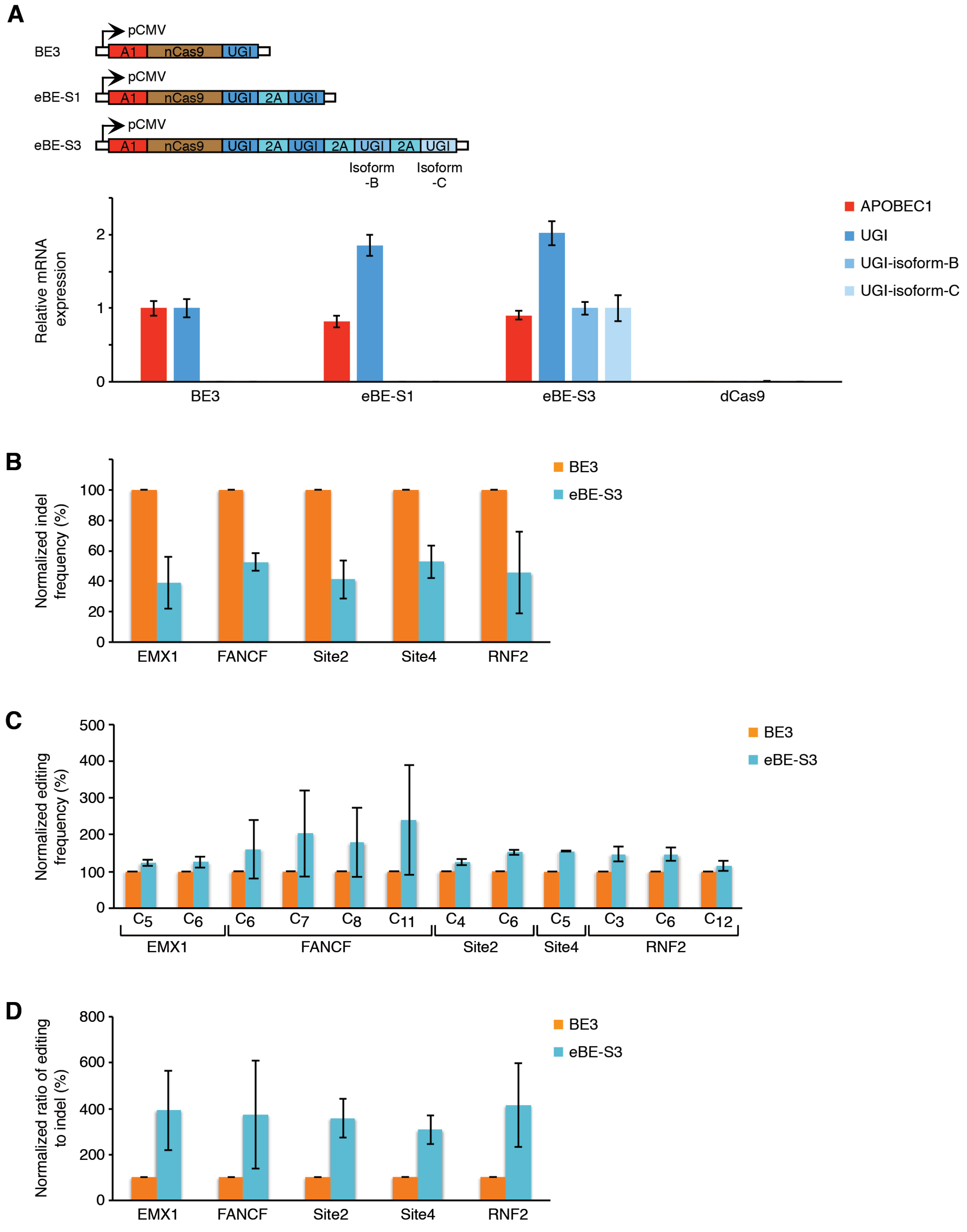

Figure S4. Enhanced base editing by eBE-S1 and eBE-S3. (A) The APOBEC1 and UGI mRNA expression levels were examined by RT-qPCR respectively. To avoid homologous recombination-caused loss of repeat sequence in plasmids, we used different

isoforms of UGI (UGI, UGI-isoform-B and UGI-isoform-C) to code for the three copies of UGI in eBE-S3. Accordingly, different primers were used for qPCR (see MATERIALS AND METHODS). (B-D) The normalized indel frequencies (B), the normalized C-to-T editing frequencies at indicated positions (C), the normalized ratios of desired editing to unwanted indels (D) were shown, setting the ones induced by BE3 as 100%.

(A-D) Error bars (±), standard deviations of 3 replicates.

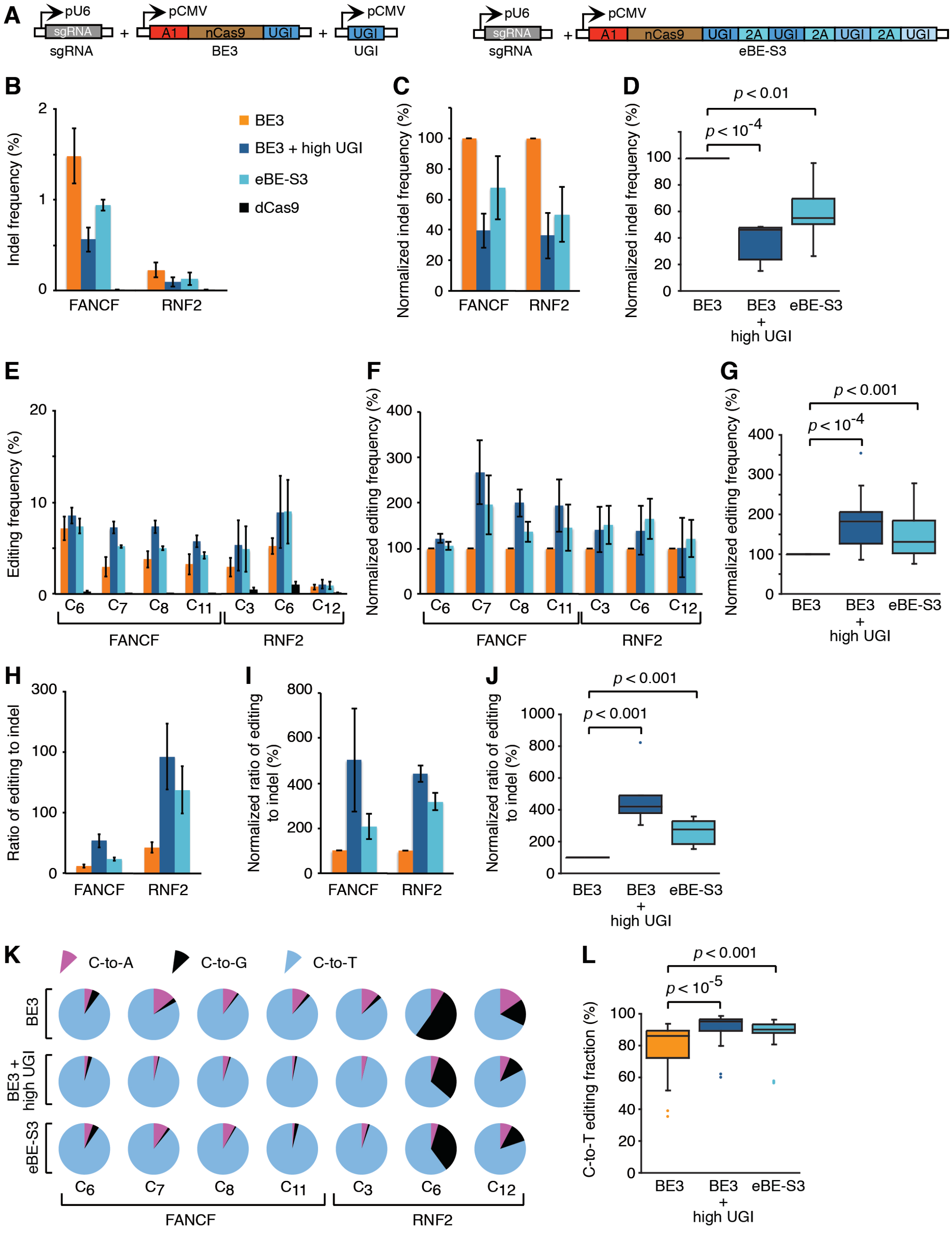

Figure S5. Performance of enhanced base editing system in HeLa cells. (A) Schematic diagram illustrating the design of sgRNA, BE3, UGI and eBE-S3 expression

vectors. The indel frequency (B), the C-to-T editing frequency at indicated position of the sgRNA target region (E), the ratio of desired editing to unwanted indels (H) and the fractions of C-to-T, C-to-A and C-to-G substitutions (K) were individually determined at the specified genomic sites for indicated conditions and plotted as following: orange represents BE3, dark blue represents BE3 + high UGI, cyan represents eBE3-S3 and black represents dCas9. The normalized indel frequency (C), the normalized C-to-T editing frequency at indicated position (F), the normalized ratio of desired editing to unwanted indels (I) were also shown, setting the ones induced by BE3 as 100%. (D, G, J, L) Statistical analyses highlighted the significant differences between BE3 (orange) and BE3 + high UGI (dark blue) or eBE-S3 (cyan) in indel frequency (D), in C-to-T editing frequency at indicated position of sgRNA target region (G), in the ratio of desired C-to-T editing to unwanted indels (J) and in the fraction of C-to-T substitution (L).

(B, C, E, F, H and I) Error bars (±), standard deviations of 3 replicates.

(D, G, J, L) *P* values, one-tailed Student's T test.
