# Supplementary material for: Enhanced base editing by co-expression of free uracil DNA glycosylase inhibitor
Source: Cell Res. 2017 Aug 29;27(10):1289–92. doi: 10.1038/cr.2017.111 (PMC5630677; doi:10.1038/cr.2017.111)
Supplement: Supplementary information, Data S1 — Materials and Methods [file cr2017111x6.pdf]

MATERIALS AND METHODS

Plasmid construction

pCMV-BE3 was obtained from Addgene (cat. no. 73021). A UGI-deleted fragment was PCR amplified by high-fidelity DNA polymerase PrimeSTAR HS (Clonetech) from pCMV-BE3 with the primer set (T2A-FOR-1/BE3-ΔUGI-R) and then fused to Nhe I/Pme I lineared pCMV-BE3 by ClonExpress® Entry One Step Cloning Kit (Vazyme) to generate the BE3-ΔUGI expressing vector pCMV-BE3-ΔUGI. UGI sequence was PCR amplified from pCMV-BE3 with the primer set (pc3.1-UGI-F/pc3.1-UGI-NLS-R) and then fused to BamH I/EcoR I linearized pcDNA3.1(+) to generate the UGI expressing vector pUGI-NLS. Oligonucleotides containing 3×Flag (pcDNA3.1-3×Flag-FOR/pcDNA3.1-3×Flag-REV) were annealed and fused to BamH I/EcoR I linearized pcDNA3.1(+) to generate pcDNA3.1-3×Flag. UGI-NLS sequence was PCR amplified from pUGI-NLS with the primer set (pc3.1-UGI-F/pc3.1-UGI-NLS-3Flag-R) and then fused to EcoR I linearized pcDNA3.1-3×Flag to generate another UGI expressing vector pUGI-NLS-3×Flag. A fragment containing Apa I/Pme I sites was amplified from pCMV-BE3 with the primer set (T2A-FOR-1/PmeI-ApaI-R) and then fused to Nhe I/PmeI linearized pCMV-BE3 to generate pCMV-BE3AP. Two fragments were amplified from pCMV-BE3 with the primer sets (T2A-FOR-1/T2A-REV-1-NEW, T2A-FOR-2/T2A-REV-2) and then fused to Nhe I/Pme I linearized pCMV-BE3 to generate pCMV-BE3T, which contains one copy of 2A-UGI sequence. The 2A-UGI sequence was then amplified from pCMV-BE3T with the primer set (ApaI-1T2AUGI-F/PmeI-1T2AUGI-R) and then ligated into the Apa I/Pme I sites of pCMV-BE3AP to generate the eBE-S1 expression vector pCMV-eBE-S1. A DNA fragment (3×2AUGI) containing three copies of 2A-UGI

was synthesized (GenScript), amplified with the primer set (ApaI-1T2AUGI-F/PmeI-3T2AUGI-R) and then ligated into the Apa I/Pme I sites of pCMV-BE3AP to generate the eBE-S3 expression vector pCMV-eBE-S3. Oligonucleotides containing sgRNA target sites were annealed and ligated into the BsaI sites of pGL3-U6-sgRNA-PGK-Puro (from Dr. Xingxu Huang's lab) to generate sgRNA-expressing vectors. Inserted sequences in expression plasmids were all validated by Sanger sequencing. The sequences of the oligos used for plasmid construction were listed in Supplementary Table 3.

Cell culture and transfection

293FT and HeLa cells from ATCC were maintained in DMEM (10566, Gibco/Thermo Fisher Scientific) + 10% FBS (16000-044, Gibco/Thermo Fisher Scientific) and have been tested to exclude mycoplasma contamination. Cells were seeded in a 6-well plate at a density of 6×10^5 per well and transfected with 500 μl serum-free Opti-MEM that contained 12 μl Lipofectamine™ 2000 (Thermo Fisher Scientific), 2.5 μg pCMV-BE3 (pCMV-eBE-S1 or pCMV-eBE-S3), 1.6 μg sgRNA-expressing plasmid without or with 50 (low), 100 (medium), or 200 (high) ng pUGI-NLS (Figure S3B). For Western blot analysis only, cells were transfected without or with 50 (low), 100 (medium), or 200 (high) ng pUGI-NLS-3×Flag (Figure S3A) to indicate the increased UGI expression at its protein level on different transfection conditions (Figure S3B). After 24 hr, puromycin (ant-pr-1, InvivoGen) was added to the media at the final concentration of 1 μg/ml. After another 48 hr, the genomic DNA was extracted from the cells with QuickExtractTM DNA Extraction Solution (QE09050, Epicentre) or the cells were lysed in 2× SDS loading buffer for western blot.

Antibodies

Primary antibodies were purchased from the following sources: against alpha-tubulin (T6199), against flag-tag (M2, F1804) for western blot - Sigma. Second antibodies were purchased from the following sources: horseradish peroxidase-conjugated goat anti-mouse (115-035-146) for western blot - Jackson Immunoresearch.

Western blotting analysis

Protein samples were incubated at 95 °C for 20 min, separated by SDS–PAGE in sample loading buffer and proteins were transferred to PVDF membranes (Thermo Scientific Pierce). After blocking with TBST (25 mM Tris pH 8.0, 150 mM NaCl, and 0.1% Tween 20) containing 5% (w/v) nonfat dry milk for 2h, the membrane was reacted overnight with indicated primary antibody. After extensive washing, the membranes were reacted with HRP-conjugated secondary antibodies for 1h. Reactive bands were detected by ECL (Thermo Scientific Pierce), and scanned by Amersham Imager 600.

RNA extraction, reverse transcription and quantitative PCR (RT-qPCR)

Total RNAs were extracted with the TransZol® Up Plus RNA Kit (TransGen, Beijing, China) according to the manufacture's instructions and reversely transcribed with cDNA Synthesis SuperMix (TransGen) according to the manufacture's instructions. Total RNA (2 μl at 500 ng/μl), 1 μl random hexamer DNA primers (0.1 μg/μl) and 5 μl nuclease-free water were heated at 65°C for 10 min and immediately put on ice, and then the reverse transcription mixture containing 10 μl 2 × TS Reaction Mix, 1 μl DNA remover, 1 μl TransScript Reverse Transcriptase/Ribonuclease Inhibitor Enzyme Mix was added. The

reactions were incubated at 25°C for 10 min, 42°C for 30 min and finally 85°C for 5 sec to inactivate the enzymes. The reactions were diluted with 80 μl of nuclease-free water. qPCR was performed with the primer set for APOBEC1 (APOBEC1-T1F: CCATGCGGCGAATGTAGTA/APOBEC1-T1R: GGCTTCATTACTCGGGCTATAA), for UGI (UGI-T1F: AGCAACTGGTTATCCAGGAATC/UGI-T1R: GTCGCTAGTCAGAAGCATGAC), for GAPDH (hGAPDH-QF: CATCAATGGAAATCCCATCA/hGAPDH-QR: TTCTCCATGGTGGTGAAGAC) and TransStart Green qPCR SuperMix (Transgen); or with the primer set for UGI_isoform-B (UGI_B-T1F: AGAACCCTGGACCTACCAA/UGI_B-T1R: TTGTTGCCGATCACTTCCTC/UGI_B-probe: 5'(6-FAM)-TGAGAAAGAGACCGGCAAACAGCT-3'(TAMRA)), for UGI_isoform-C (UGI_C-T1F: TCTTATGTTGCCTGAGGAAGTC/UGI_C-T1R: CGCCCATGGTTTGTACTCT/UGI_C-probe: 5'(6-FAM)-ACCGCTTACGATGAGTCCACAGATG-3'(TAMRA)) and TransStart Probe qPCR SuperMix (Transgen) according to the instruction.

DNA library preparation and sequencing

Target genomic sequences were PCR amplified by high-fidelity DNA polymerase PrimeSTAR HS (Clonetech) with primers flanking each examined sgRNA target site. The sgRNA target sites and PCR primers used to amplify target genomic sequences were listed in Supplementary Table 4. Indexed DNA libraries were prepared by using the TruSeq ChIP Sample Preparation Kit (Illumina) with some minor modifications. Briefly, the PCR products amplified from genomic DNA regions were fragmented by Covaris S220. The fragmented DNAs were then PCR amplified by using the TruSeq ChIP Sample

Preparation Kit (Illumina). After quantitated with Qubit High-Sensitivity DNA kit (Invitrogen), PCR products with different tags were pooled together for deep sequencing by using the Illumina Hiseq 2500 (1×100), Hiseq X-10 (2×150) or NextSeq 500 (2X150) at CAS-MPG Partner Institute for Computational Biology Omics Core, Shanghai, China. Raw read qualities were evaluated by FastQC (http://www.bioinformatics.babraham.ac.uk/projects/fastqc/). For paired end sequencing, only R1 reads were used. Adaptor sequences and read sequences on both ends with Phred quality score lower than 30 were trimmed. Trimmed reads were then mapped with the BWA-MEM algorithm (BWA v0.7.9a) to target sequences. After piped up with samtools (v0.1.18), indels and base substitutions were further calculated.

Indel frequency calculation

Indels were estimated in the aligned regions spanning from upstream eight nucleotides to the target site to downstream 19 nucleotides to PAM sites (50 bp). Indel frequencies were subsequently calculated by dividing reads containing at least two inserted and/or deleted nucleotides by all the mapped reads at the same region. Counts of indel-containing reads and total mapped reads are listed in Supplementary Table 1. Indel frequencies were calculated by dividing indel-containing reads by total mapped reads.

Base substitution calculation

Base substitutions were selected at each position of the examined sgRNA target sites that mapped with at least 500 independent reads, and obvious base substitutions were only observed at the targeted base editing sites. Counts of reads for each base and total reads

are listed in Supplementary Table 2. Base substitution frequencies were calculated by dividing base substitution reads by total reads. For each sgRNA, the ratio of C-to-T base substitution over indel was calculated by dividing the sum of C-to-T base substitution frequencies at all editing sites by the indel frequency of 50-bp region around sgRNA target site (from upstream eight nucleotides to the target site to downstream 19 nucleotides to PAM sites).

Data Availability

Deep sequencing data can be accessed at the NCBI Gene Expression Omnibus (accession no: GSE98685, https://www.ncbi.nlm.nih.gov/geo/query/acc.cgi?acc=GSE98685).

DNA Sequence

pCMV-BE3-ΔUGI

ATGAGCTCAGAGACTGGCCCAGTGGCTGTGGACCCCACATTGAGACGGCGGATCGAGCCCCATGAGTTTGAGGTATTCTTCGATCCGAGA
GAGCTCCGCAAGGAGACCTGCCTGCTTTACGAAATTAATTGGGGGGGCCGGCACTCCATTTGGCGACATACATCACAGAACACTAACAAG
CACGTCGAAGTCAACTTCATCGAGAAGTTCACGACAGAAAGATATTTCTGTCCGAACACAAGGTGCAGCATTACCTGGTTTCTCAGCTGG
AGCCCATGCGGCGAATGTAGTAGGGCCATCACTGAATTCCTGTCAAGGTATCCCCACGTCACTCTGTTTATTTACATCGCAAGGCTGTAC
CACCACGCTGACCCCCGCAATCGACAAGGCCTGCGGGATTTGATCTCTTCAGGTGTGACTATCCAAATTATGACTGAGCAGGAGTCAGGA
TACTGCTGGAGAAACTTTGTGAATTATAGCCCGAGTAATGAAGCCCACTGGCCTAGGTATCCCCATCTGTGGGTACGACTGTACGTTCTT
GAACTGTACTGCATCATACTGGGCCTGCCTCCTTGTCTCAACATTCTGAGAAGGAAGCAGCCACAGCTGACATTCTTTACCATCGCTCTT
CAGTCTTGTCATTACCAGCGACTGCCCCCACACATTCTCTGGGCCACCGGGTTGAAAagcggcagcgagactcccgggacctcagagtccgccacacccg
aaagtGATAAAAAGTATTCTATTGGTTTAGCCATCGGCACTAATTCCGTTGGATGGGCTGTCATAACCGATGAATACAAAGTACCTTCAAA
GAAATTTAAGGTGTTGGGGAACACAGACCGTCATTCGATTAAAAAGAATCTTATCGGTGCCCTCCTATTCGATAGTGGCGAAACGGCAG
AGGCGACTCGCCTGAAACGAACCGCTCGGAGAAGGTATACACGTCGCAAGAACCGAATATGTTACTTACAAGAAATTTTTAGCAATGAG
ATGGCCAAAGTTGACGATTCTTTCTTTCACCGTTTGGAAGAGTCCTTCCTTGTCGAAGAGGACAAGAAACATGAACGGCACCCCATCTTT
GGAAACATAGTAGATGAGGTGGCATATCATGAAAAGTACCCAACGATTTATCACCTCAGAAAAAAGCTAGTTGACTCAACTGATAAAGC
GGACCTGAGGTTAATCTACTTGGCTCTTGCCCATATGATAAAGTTCCGTGGGCACTTTCTCATTGAGGGTGATCTAAATCCGGACAACTC
GGATGTCGACAAACTGTTCATCCAGTTAGTACAAACCTATAATCAGTTGTTTGAAGAGAACCCTATAAATGCAAGTGGCGTGGATGCGA
AGGCTATTCTTAGCGCCCGCCTCTCTAAATCCCGACGGCTAGAAAACCTGATCGCACAATTACCCGGAGAGAAGAAAAATGGGTTGTTCG
GTAACCTTATAGCGCTCTCACTAGGCCTGACACCAAATTTTAAGTCGAACTTCGACTTAGCTGAAGATGCCAAATTGCAGCTTAGTAAGG
ACACGTACGATGACGATCTCGACAATCTACTGGCACAAATTGGAGATCAGTATGCGGACTTATTTTTGGCTGCCAAAAACCTTAGCGATG
CAATCCTCCTATCTGACATACTGAGAGTTAATACTGAGATTACCAAGGCGCCGTTATCCGCTTCAATGATCAAAAGGTACGATGAACATC
ACCAAGACTTGACACTTCTCAAGGCCCTAGTCCGTCAGCAACTGCCTGAGAAATATAAGGAAATATTCTTTGATCAGTCGAAAAACGGGT
ACGCAGGTTATATTGACGGCGGAGCGAGTCAAGAGGAATTCTACAAGTTTATCAAACCCATATTAGAGAAGATGGATGGGACGGAAGAG
TTGCTTGTAAAACTCAATCGCGAAGATCTACTGCGAAAGCAGCGGACTTTCGACAACGGTAGCATTCCACATCAAATCCACTTAGGCGAA
TTGCATGCTATACTTAGAAGGCAGGAGGATTTTTATCCGTTCCTCAAAGACAATCGTGAAAAGATTGAGAAAATCCTAACCTTTCGCAT
ACCTTACTATGTGGGACCCCTGGCCCGAGGGAACTCTCGGTTCGCATGGATGACAAGAAAGTCCGAAGAAACGATTACTCCATGGAATTT
TGAGGAAGTTGTCGATAAAGGTGCGTCAGCTCAATCGTTCATCGAGAGGATGACCAACTTTGACAAGAATTTACCGAACGAAAAAGTAT
TGCCTAAGCACAGTTTACTTTACGAGTATTTCACAGTGTACAATGAACTCACGAAAGTTAAGTATGTCACTGAGGGCATGCGTAAACCCG
CCTTTCTAAGCGGAGAACAGAAGAAAGCAATAGTAGATCTGTTATTCAAGACCAACCGCAAAGTGACAGTTAAGCAATTGAAAGAGGAC
TACTTTAAGAAAATTGAATGCTTCGATTCTGTCGAGATCTCCGGGGTAGAAGATCGATTTAATGCGTCACTTGGTACGTATCATGACCTC

CTAAAGATAATTAAAGATAAGGACTTCCTGGATAACGAAGAGAATGAAGATATCTTAGAAGATATAGTGTTGACTCTTACCCTCTTTGA
AGATCGGGAAATGATTGAGGAAAGACTAAAAACATACGCTCACCTGTTCGACGATAAGGTTATGAAACAGTTAAAGAGGCGTCGCTATA
CGGGCTGGGGACGATTGTCGCGGAAACTTATCAACGGGATAAGAGACAAGCAAAGTGGTAAAACTATTCTCGATTTTCTAAAGAGCGAC
GGCTTCGCCAATAGGAACTTTATGCAGCTGATCCATGATGACTCTTTAACCTTCAAAGAGGATATACAAAAGGCACAGGTTTCCGGACAA
GGGGACTCATTGCACGAACATATTGCGAATCTTGCTGGTTCGCCAGCCATCAAAAAGGGCATACTCCAGACAGTCAAAGTAGTGGATGAG
CTAGTTAAGGTCATGGGACGTCACAAACCGGAAAACATTGTAATCGAGATGGCACGCGAAAATCAAACGACTCAGAAGGGGCAAAAAAA
CAGTCGAGAGCGGATGAAGAGAATAGAAGAGGGTATTAAAGAACTGGGCAGCCAGATCTTAAAGGAGCATCCTGTGGAAAATACCCAAT
TGCAGAACGAGAAACTTTACCTCTATTACCTACAAAATGGAAGGGACATGTATGTTGATCAGGAACTGGACATAAACCGTTTATCTGAT
TACGACGTCGATCACATTGTACCCCAATCCTTTTTGAAGGACGATTCAATCGACAATAAAGTGCTTACACGCTCGGATAAGAACCGAGGG
AAAAGTGACAATGTTCCAAGCGAGGAAGTCGTAAAGAAAATGAAGAACTATTGGCGGCAGCTCCTAAATGCGAAACTGATAACGCAAAG
AAAGTTCGATAACTTAACTAAAGCTGAGAGGGGTGGCTTGTCTGAACTTGACAAGGCCGGATTTATTAAACGTCAGCTCGTGGAAACCC
GCCAAATCACAAAGCATGTTGCACAGATACTAGATTCCCGAATGAATACGAAATACGACGAGAACGATAAGCTGATTCGGGAAGTCAAA
GTAATCACTTTAAAGTCAAAATTGGTGTCGGACTTCAGAAAGGATTTTCAATTCTATAAAGTTAGGGAGATAAATAACTACCACCATGC
GCACGACGCTTATCTTAATGCCGTCGTAGGGACCGCACTCATTAAGAAATACCCGAAGCTAGAAAGTGAGTTTGTGTATGGTGATTACA
AAGTTTATGACGTCCGTAAGATGATCGCGAAAAGCGAACAGGAGATAGGCAAGGCTACAGCCAAATACTTCTTTTATTCTAACATTATG
AATTTCTTTAAGACGGAAATCACTCTGGCAAACGGAGAGATACGCAAACGACCTTTAATTGAAACCAATGGGGAGACAGGTGAAATCGT
ATGGGATAAGGGCCGGGACTTCGCGACGGTGAGAAAAGTTTTGTCCATGCCCCAAGTCAACATAGTAAAGAAAACTGAGGTGCAGACCG
GAGGGTTTTCAAAGGAATCGATTCTTCCAAAAAGGAATAGTGATAAGCTCATCGCTCGTAAAAAGGACTGGGACCCGAAAAAGTACGGT
GGCTTCGATAGCCCTACAGTTGCCTATTCTGTCCTAGTAGTGGCAAAAGTTGAGAAGGGAAAATCCAAGAAACTGAAGTCAGTCAAAGA
ATTATTGGGGATAACGATTATGGAGCGCTCGTCTTTTGAAAAGAACCCCATCGACTTCCTTGAGGCGAAAGGTTACAAGGAAGTAAAAA
AGGATCTCATAATTAAACTACCAAAGTATAGTCTGTTTGAGTTAGAAAATGGCCGAAAACGGATGTTGGCTAGCGCCGGAGAGCTTCAA
AAGGGGAACGAACTCGCACTACCGTCTAAATACGTGAATTTCCTGTATTTAGCGTCCCATTACGAGAAGTTGAAAGGTTCACCTGAAGAT
AACGAACAGAAGCAACTTTTTGTTGAGCAGCACAAACATTATCTCGACGAAATCATAGAGCAAATTTCGGAATTCAGTAAGAGAGTCAT
CCTAGCTGATGCCAATCTGGACAAAGTATTAAGCGCATACAACAAGCACAGGGATAAACCCATACGTGAGCAGGCGGAAAATATTATCC
ATTTGTTTACTCTTACCAACCTCGGCGCTCCAGCCGCATTCAAGTATTTTGACACAACGATAGATCGCAAACGATACACTTCTACCAAGG
AGGTGCTAGACGCGACACTGATTCACCAATCCATCACGGGATTATATGAAACTCGGATAGATTTGTCACAGCTTGGGGGTGACtctggtggtt
ctCCCAAGAAGAAGAGGAAAGTC**TGA**

pUGI-NLS:

ATGACTAATCTGTCAGATATTATTGAAAAGGAGACCGGTAAGCAACTGGTTATCCAGGAATCCATCCTCATGCTCCCAGAGGAGGTGGA
AGAAGTCATTGGGAACAAGCCGGAAAGCGATATACTCGTGCACACCGCCTACGACGAGAGCACCGACGAGAATGTCATGCTTCTGACTAG
CGACGCCCCTGAATACAAGCCTTGGGCTCTGGTCATACAGGATAGCAACGGTGAGAACAAGATTAAGATGCTCCCCAAGAAGAAGAGGA
AAGTC**TAG**

pUGI-NLS-3×Flag:

ATGACTAATCTGTCAGATATTATTGAAAAGGAGACCGGTAAGCAACTGGTTATCCAGGAATCCATCCTCATGCTCCCAGAGGAGGTGGA
AGAAGTCATTGGGAACAAGCCGGAAAGCGATATACTCGTGCACACCGCCTACGACGAGAGCACCGACGAGAATGTCATGCTTCTGACTAG
CGACGCCCCTGAATACAAGCCTTGGGCTCTGGTCATACAGGATAGCAACGGTGAGAACAAGATTAAGATGCTCCCCAAGAAGAAGAGGA
AAGTCGACTACAAAGACCATGACGGTGATTATAAAGATCATGACATCGATTACAAGGATGACGATGACAAG**TAG**

pCMV-eBE-S1:

ATGAGCTCAGAGACTGGCCCAGTGGCTGTGGACCCCACATTGAGACGGCGGATCGAGCCCCATGAGTTTGAGGTATTCTTCGATCCGAGA
GAGCTCCGCAAGGAGACCTGCCTGCTTTACGAAATTAATTGGGGGGGCCGGCACTCCATTTGGCGACATACATCACAGAACACTAACAAG
CACGTCGAAGTCAACTTCATCGAGAAGTTCACGACAGAAAGATATTTCTGTCCGAACACAAGGTGCAGCATTACCTGGTTTCTCAGCTGG
AGCCCATGCGGCGAATGTAGTAGGGCCATCACTGAATTCCTGTCAAGGTATCCCCACGTCACTCTGTTTATTTACATCGCAAGGCTGTAC
CACCACGCTGACCCCCGCAATCGACAAGGCCTGCGGGATTTGATCTCTTCAGGTGTGACTATCCAAATTATGACTGAGCAGGAGTCAGGA
TACTGCTGGAGAAACTTTGTGAATTATAGCCCGAGTAATGAAGCCCACTGGCCTAGGTATCCCCATCTGTGGGTACGACTGTACGTTCTT
GAACTGTACTGCATCATACTGGGCCTGCCTCCTTGTCTCAACATTCTGAGAAGGAAGCAGCCACAGCTGACATTCTTTACCATCGCTCTT
CAGTCTTGTCATTACCAGCGACTGCCCCCACACATTCTCTGGGCCACCGGGTTGAAAagcggcagcgagactcccgggacctcagagtccgccacacccg
aaagtGATAAAAAGTATTCTATTGGTTTAGCCATCGGCACTAATTCCGTTGGATGGGCTGTCATAACCGATGAATACAAAGTACCTTCAAA
GAAATTTAAGGTGTTGGGGAACACAGACCGTCATTCGATTAAAAAGAATCTTATCGGTGCCCTCCTATTCGATAGTGGCGAAACGGCAG
AGGCGACTCGCCTGAAACGAACCGCTCGGAGAAGGTATACACGTCGCAAGAACCGAATATGTTACTTACAAGAAATTTTTAGCAATGAG
ATGGCCAAAGTTGACGATTCTTTCTTTCACCGTTTGGAAGAGTCCTTCCTTGTCGAAGAGGACAAGAAACATGAACGGCACCCCATCTTT
GGAAACATAGTAGATGAGGTGGCATATCATGAAAAGTACCCAACGATTTATCACCTCAGAAAAAAGCTAGTTGACTCAACTGATAAAGC

GGACCTGAGGTTAATCTACTTGGCTCTTGCCCATATGATAAAGTTCCGTGGGCACTTTCTCATTGAGGGTGATCTAAATCCGGACAACTC
GGATGTCGACAAACTGTTCATCCAGTTAGTACAAACCTATAATCAGTTGTTTGAAGAGAACCCTATAAATGCAAGTGGCGTGGATGCGA
AGGCTATTCTTAGCGCCCGCCTCTCTAAATCCCGACGGCTAGAAAACCTGATCGCACAATTACCCGGAGAGAAGAAAAATGGGTTGTTCG
GTAACCTTATAGCGCTCTCACTAGGCCTGACACCAAATTTTAAGTCGAACTTCGACTTAGCTGAAGATGCCAAATTGCAGCTTAGTAAGG
ACACGTACGATGACGATCTCGACAATCTACTGGCACAAATTGGAGATCAGTATGCGGACTTATTTTTGGCTGCCAAAAACCTTAGCGATG
CAATCCTCCTATCTGACATACTGAGAGTTAATACTGAGATTACCAAGGCGCCGTTATCCGCTTCAATGATCAAAAGGTACGATGAACATC
ACCAAGACTTGACACTTCTCAAGGCCCTAGTCCGTCAGCAACTGCCTGAGAAATATAAGGAAATATTCTTTGATCAGTCGAAAAACGGGT
ACGCAGGTTATATTGACGGCGGAGCGAGTCAAGAGGAATTCTACAAGTTTATCAAACCCATATTAGAGAAGATGGATGGGACGGAAGAG
TTGCTTGTAAAACTCAATCGCGAAGATCTACTGCGAAAGCAGCGGACTTTCGACAACGGTAGCATTCCACATCAAATCCACTTAGGCGAA
TTGCATGCTATACTTAGAAGGCAGGAGGATTTTTATCCGTTCCTCAAAGACAATCGTGAAAAGATTGAGAAAATCCTAACCTTTCGCAT
ACCTTACTATGTGGGACCCCTGGCCCGAGGGAACTCTCGGTTCGCATGGATGACAAGAAAGTCCGAAGAAACGATTACTCCATGGAATTT
TGAGGAAGTTGTCGATAAAGGTGCGTCAGCTCAATCGTTCATCGAGAGGATGACCAACTTTGACAAGAATTTACCGAACGAAAAAGTAT
TGCCTAAGCACAGTTTACTTTACGAGTATTTCACAGTGTACAATGAACTCACGAAAGTTAAGTATGTCACTGAGGGCATGCGTAAACCCG
CCTTTCTAAGCGGAGAACAGAAGAAAGCAATAGTAGATCTGTTATTCAAGACCAACCGCAAAGTGACAGTTAAGCAATTGAAAGAGGAC
TACTTTAAGAAAATTGAATGCTTCGATTCTGTCGAGATCTCCGGGGTAGAAGATCGATTTAATGCGTCACTTGGTACGTATCATGACCTC
CTAAAGATAATTAAAGATAAGGACTTCCTGGATAACGAAGAGAATGAAGATATCTTAGAAGATATAGTGTTGACTCTTACCCTCTTTGA
AGATCGGGAAATGATTGAGGAAAGACTAAAAACATACGCTCACCTGTTCGACGATAAGGTTATGAAACAGTTAAAGAGGCGTCGCTATA
CGGGCTGGGGACGATTGTCGCGGAAACTTATCAACGGGATAAGAGACAAGCAAAGTGGTAAAACTATTCTCGATTTTCTAAAGAGCGAC
GGCTTCGCCAATAGGAACTTTATGCAGCTGATCCATGATGACTCTTTAACCTTCAAAGAGGATATACAAAAGGCACAGGTTTCCGGACAA
GGGGACTCATTGCACGAACATATTGCGAATCTTGCTGGTTCGCCAGCCATCAAAAAGGGCATACTCCAGACAGTCAAAGTAGTGGATGAG
CTAGTTAAGGTCATGGGACGTCACAAACCGGAAAACATTGTAATCGAGATGGCACGCGAAAATCAAACGACTCAGAAGGGGCAAAAAAA
CAGTCGAGAGCGGATGAAGAGAATAGAAGAGGGTATTAAAGAACTGGGCAGCCAGATCTTAAAGGAGCATCCTGTGGAAAATACCCAAT
TGCAGAACGAGAAACTTTACCTCTATTACCTACAAAATGGAAGGGACATGTATGTTGATCAGGAACTGGACATAAACCGTTTATCTGAT
TACGACGTCGATCACATTGTACCCCAATCCTTTTTGAAGGACGATTCAATCGACAATAAAGTGCTTACACGCTCGGATAAGAACCGAGGG
AAAAGTGACAATGTTCCAAGCGAGGAAGTCGTAAAGAAAATGAAGAACTATTGGCGGCAGCTCCTAAATGCGAAACTGATAACGCAAAG
AAAGTTCGATAACTTAACTAAAGCTGAGAGGGGTGGCTTGTCTGAACTTGACAAGGCCGGATTTATTAAACGTCAGCTCGTGGAAACCC
GCCAAATCACAAAGCATGTTGCACAGATACTAGATTCCCGAATGAATACGAAATACGACGAGAACGATAAGCTGATTCGGGAAGTCAAA
GTAATCACTTTAAAGTCAAAATTGGTGTCGGACTTCAGAAAGGATTTTCAATTCTATAAAGTTAGGGAGATAAATAACTACCACCATGC
GCACGACGCTTATCTTAATGCCGTCGTAGGGACCGCACTCATTAAGAAATACCCGAAGCTAGAAAGTGAGTTTGTGTATGGTGATTACA
AAGTTTATGACGTCCGTAAGATGATCGCGAAAAGCGAACAGGAGATAGGCAAGGCTACAGCCAAATACTTCTTTTATTCTAACATTATG
AATTTCTTTAAGACGGAAATCACTCTGGCAAACGGAGAGATACGCAAACGACCTTTAATTGAAACCAATGGGGAGACAGGTGAAATCGT
ATGGGATAAGGGCCGGGACTTCGCGACGGTGAGAAAAGTTTTGTCCATGCCCCAAGTCAACATAGTAAAGAAAACTGAGGTGCAGACCG
GAGGGTTTTCAAAGGAATCGATTCTTCCAAAAAGGAATAGTGATAAGCTCATCGCTCGTAAAAAGGACTGGGACCCGAAAAAGTACGGT
GGCTTCGATAGCCCTACAGTTGCCTATTCTGTCCTAGTAGTGGCAAAAGTTGAGAAGGGAAAATCCAAGAAACTGAAGTCAGTCAAAGA
ATTATTGGGGATAACGATTATGGAGCGCTCGTCTTTTGAAAAGAACCCCATCGACTTCCTTGAGGCGAAAGGTTACAAGGAAGTAAAAA
AGGATCTCATAATTAAACTACCAAAGTATAGTCTGTTTGAGTTAGAAAATGGCCGAAAACGGATGTTGGCTAGCGCCGGAGAGCTTCAA
AAGGGGAACGAACTCGCACTACCGTCTAAATACGTGAATTTCCTGTATTTAGCGTCCCATTACGAGAAGTTGAAAGGTTCACCTGAAGAT
AACGAACAGAAGCAACTTTTTGTTGAGCAGCACAAACATTATCTCGACGAAATCATAGAGCAAATTTCGGAATTCAGTAAGAGAGTCAT
CCTAGCTGATGCCAATCTGGACAAAGTATTAAGCGCATACAACAAGCACAGGGATAAACCCATACGTGAGCAGGCGGAAAATATTATCC
ATTTGTTTACTCTTACCAACCTCGGCGCTCCAGCCGCATTCAAGTATTTTGACACAACGATAGATCGCAAACGATACACTTCTACCAAGG
AGGTGCTAGACGCGACACTGATTCACCAATCCATCACGGGATTATATGAAACTCGGATAGATTTGTCACAGCTTGGGGGTGACtctggtggtt
ctACTAATCTGTCAGATATTATTGAAAAGGAGACCGGTAAGCAACTGGTTATCCAGGAATCCATCCTCATGCTCCCAGAGGAGGTGGAAG
AAGTCATTGGGAACAAGCCGGAAAGCGATATACTCGTGCACACCGCCTACGACGAGAGCACCGACGAGAATGTCATGCTTCTGACTAGCG
ACGCCCCTGAATACAAGCCTTGGGCTCTGGTCATACAGGATAGCAACGGTGAGAACAAGATTAAGATGCTCtctggtggttctCCCAAGAAGA
AGAGGAAAGTCgggcccGAGGGCAGAGGAAGTCTGCTAACATGCGGTGACGTCGAGGAGAATCCTGGCCCAACTAATCTGTCAGATATTAT
TGAAAAGGAGACCGGTAAGCAACTGGTTATCCAGGAATCCATCCTCATGCTCCCAGAGGAGGTGGAAGAAGTCATTGGGAACAAGCCGG
AAAGCGATATACTCGTGCACACCGCCTACGACGAGAGCACCGACGAGAATGTCATGCTTCTGACTAGCGACGCCCCTGAATACAAGCCTT
GGGCTCTGGTCATACAGGATAGCAACGGTGAGAACAAGATTAAGATGCTCtctggtggttctCCCAAGAAGAAGAGGAAAGTC**TAA**

pCMV-eBE-S3:

ATGAGCTCAGAGACTGGCCCAGTGGCTGTGGACCCCACATTGAGACGGCGGATCGAGCCCCATGAGTTTGAGGTATTCTTCGATCCGAGA
GAGCTCCGCAAGGAGACCTGCCTGCTTTACGAAATTAATTGGGGGGGCCGGCACTCCATTTGGCGACATACATCACAGAACACTAACAAG
CACGTCGAAGTCAACTTCATCGAGAAGTTCACGACAGAAAGATATTTCTGTCCGAACACAAGGTGCAGCATTACCTGGTTTCTCAGCTGG
AGCCCATGCGGCGAATGTAGTAGGGCCATCACTGAATTCCTGTCAAGGTATCCCCACGTCACTCTGTTTATTTACATCGCAAGGCTGTAC
CACCACGCTGACCCCCGCAATCGACAAGGCCTGCGGGATTTGATCTCTTCAGGTGTGACTATCCAAATTATGACTGAGCAGGAGTCAGGA
TACTGCTGGAGAAACTTTGTGAATTATAGCCCGAGTAATGAAGCCCACTGGCCTAGGTATCCCCATCTGTGGGTACGACTGTACGTTCTT
GAACTGTACTGCATCATACTGGGCCTGCCTCCTTGTCTCAACATTCTGAGAAGGAAGCAGCCACAGCTGACATTCTTTACCATCGCTCTT
CAGTCTTGTCATTACCAGCGACTGCCCCCACACATTCTCTGGGCCACCGGGTTGAAAagcggcagcgagactcccgggacctcagagtccgccacacccg
aaagtGATAAAAAGTATTCTATTGGTTTAGCCATCGGCACTAATTCCGTTGGATGGGCTGTCATAACCGATGAATACAAAGTACCTTCAAA
GAAATTTAAGGTGTTGGGGAACACAGACCGTCATTCGATTAAAAAGAATCTTATCGGTGCCCTCCTATTCGATAGTGGCGAAACGGCAG
AGGCGACTCGCCTGAAACGAACCGCTCGGAGAAGGTATACACGTCGCAAGAACCGAATATGTTACTTACAAGAAATTTTTAGCAATGAG

ATGGCCAAAGTTGACGATTCTTTCTTTCACCGTTTGGAAGAGTCCTTCCTTGTCGAAGAGGACAAGAAACATGAACGGCACCCCATCTTT
GGAAACATAGTAGATGAGGTGGCATATCATGAAAAGTACCCAACGATTTATCACCTCAGAAAAAAGCTAGTTGACTCAACTGATAAAGC
GGACCTGAGGTTAATCTACTTGGCTCTTGCCCATATGATAAAGTTCCGTGGGCACTTTCTCATTGAGGGTGATCTAAATCCGGACAACTC
GGATGTCGACAAACTGTTCATCCAGTTAGTACAAACCTATAATCAGTTGTTTGAAGAGAACCCTATAAATGCAAGTGGCGTGGATGCGA
AGGCTATTCTTAGCGCCCGCCTCTCTAAATCCCGACGGCTAGAAAACCTGATCGCACAATTACCCGGAGAGAAGAAAAATGGGTTGTTCG
GTAACCTTATAGCGCTCTCACTAGGCCTGACACCAAATTTTAAGTCGAACTTCGACTTAGCTGAAGATGCCAAATTGCAGCTTAGTAAGG
ACACGTACGATGACGATCTCGACAATCTACTGGCACAAATTGGAGATCAGTATGCGGACTTATTTTTGGCTGCCAAAAACCTTAGCGATG
CAATCCTCCTATCTGACATACTGAGAGTTAATACTGAGATTACCAAGGCGCCGTTATCCGCTTCAATGATCAAAAGGTACGATGAACATC
ACCAAGACTTGACACTTCTCAAGGCCCTAGTCCGTCAGCAACTGCCTGAGAAATATAAGGAAATATTCTTTGATCAGTCGAAAAACGGGT
ACGCAGGTTATATTGACGGCGGAGCGAGTCAAGAGGAATTCTACAAGTTTATCAAACCCATATTAGAGAAGATGGATGGGACGGAAGAG
TTGCTTGTAAAACTCAATCGCGAAGATCTACTGCGAAAGCAGCGGACTTTCGACAACGGTAGCATTCCACATCAAATCCACTTAGGCGAA
TTGCATGCTATACTTAGAAGGCAGGAGGATTTTTATCCGTTCCTCAAAGACAATCGTGAAAAGATTGAGAAAATCCTAACCTTTCGCAT
ACCTTACTATGTGGGACCCCTGGCCCGAGGGAACTCTCGGTTCGCATGGATGACAAGAAAGTCCGAAGAAACGATTACTCCATGGAATTT
TGAGGAAGTTGTCGATAAAGGTGCGTCAGCTCAATCGTTCATCGAGAGGATGACCAACTTTGACAAGAATTTACCGAACGAAAAAGTAT
TGCCTAAGCACAGTTTACTTTACGAGTATTTCACAGTGTACAATGAACTCACGAAAGTTAAGTATGTCACTGAGGGCATGCGTAAACCCG
CCTTTCTAAGCGGAGAACAGAAGAAAGCAATAGTAGATCTGTTATTCAAGACCAACCGCAAAGTGACAGTTAAGCAATTGAAAGAGGAC
TACTTTAAGAAAATTGAATGCTTCGATTCTGTCGAGATCTCCGGGGTAGAAGATCGATTTAATGCGTCACTTGGTACGTATCATGACCTC
CTAAAGATAATTAAAGATAAGGACTTCCTGGATAACGAAGAGAATGAAGATATCTTAGAAGATATAGTGTTGACTCTTACCCTCTTTGA
AGATCGGGAAATGATTGAGGAAAGACTAAAAACATACGCTCACCTGTTCGACGATAAGGTTATGAAACAGTTAAAGAGGCGTCGCTATA
CGGGCTGGGGACGATTGTCGCGGAAACTTATCAACGGGATAAGAGACAAGCAAAGTGGTAAAACTATTCTCGATTTTCTAAAGAGCGAC
GGCTTCGCCAATAGGAACTTTATGCAGCTGATCCATGATGACTCTTTAACCTTCAAAGAGGATATACAAAAGGCACAGGTTTCCGGACAA
GGGGACTCATTGCACGAACATATTGCGAATCTTGCTGGTTCGCCAGCCATCAAAAAGGGCATACTCCAGACAGTCAAAGTAGTGGATGAG
CTAGTTAAGGTCATGGGACGTCACAAACCGGAAAACATTGTAATCGAGATGGCACGCGAAAATCAAACGACTCAGAAGGGGCAAAAAAA
CAGTCGAGAGCGGATGAAGAGAATAGAAGAGGGTATTAAAGAACTGGGCAGCCAGATCTTAAAGGAGCATCCTGTGGAAAATACCCAAT
TGCAGAACGAGAAACTTTACCTCTATTACCTACAAAATGGAAGGGACATGTATGTTGATCAGGAACTGGACATAAACCGTTTATCTGAT
TACGACGTCGATCACATTGTACCCCAATCCTTTTTGAAGGACGATTCAATCGACAATAAAGTGCTTACACGCTCGGATAAGAACCGAGGG
AAAAGTGACAATGTTCCAAGCGAGGAAGTCGTAAAGAAAATGAAGAACTATTGGCGGCAGCTCCTAAATGCGAAACTGATAACGCAAAG
AAAGTTCGATAACTTAACTAAAGCTGAGAGGGGTGGCTTGTCTGAACTTGACAAGGCCGGATTTATTAAACGTCAGCTCGTGGAAACCC
GCCAAATCACAAAGCATGTTGCACAGATACTAGATTCCCGAATGAATACGAAATACGACGAGAACGATAAGCTGATTCGGGAAGTCAAA
GTAATCACTTTAAAGTCAAAATTGGTGTCGGACTTCAGAAAGGATTTTCAATTCTATAAAGTTAGGGAGATAAATAACTACCACCATGC
GCACGACGCTTATCTTAATGCCGTCGTAGGGACCGCACTCATTAAGAAATACCCGAAGCTAGAAAGTGAGTTTGTGTATGGTGATTACA
AAGTTTATGACGTCCGTAAGATGATCGCGAAAAGCGAACAGGAGATAGGCAAGGCTACAGCCAAATACTTCTTTTATTCTAACATTATG
AATTTCTTTAAGACGGAAATCACTCTGGCAAACGGAGAGATACGCAAACGACCTTTAATTGAAACCAATGGGGAGACAGGTGAAATCGT
ATGGGATAAGGGCCGGGACTTCGCGACGGTGAGAAAAGTTTTGTCCATGCCCCAAGTCAACATAGTAAAGAAAACTGAGGTGCAGACCG
GAGGGTTTTCAAAGGAATCGATTCTTCCAAAAAGGAATAGTGATAAGCTCATCGCTCGTAAAAAGGACTGGGACCCGAAAAAGTACGGT
GGCTTCGATAGCCCTACAGTTGCCTATTCTGTCCTAGTAGTGGCAAAAGTTGAGAAGGGAAAATCCAAGAAACTGAAGTCAGTCAAAGA
ATTATTGGGGATAACGATTATGGAGCGCTCGTCTTTTGAAAAGAACCCCATCGACTTCCTTGAGGCGAAAGGTTACAAGGAAGTAAAAA
AGGATCTCATAATTAAACTACCAAAGTATAGTCTGTTTGAGTTAGAAAATGGCCGAAAACGGATGTTGGCTAGCGCCGGAGAGCTTCAA
AAGGGGAACGAACTCGCACTACCGTCTAAATACGTGAATTTCCTGTATTTAGCGTCCCATTACGAGAAGTTGAAAGGTTCACCTGAAGAT
AACGAACAGAAGCAACTTTTTGTTGAGCAGCACAAACATTATCTCGACGAAATCATAGAGCAAATTTCGGAATTCAGTAAGAGAGTCAT
CCTAGCTGATGCCAATCTGGACAAAGTATTAAGCGCATACAACAAGCACAGGGATAAACCCATACGTGAGCAGGCGGAAAATATTATCC
ATTTGTTTACTCTTACCAACCTCGGCGCTCCAGCCGCATTCAAGTATTTTGACACAACGATAGATCGCAAACGATACACTTCTACCAAGG
AGGTGCTAGACGCGACACTGATTCACCAATCCATCACGGGATTATATGAAACTCGGATAGATTTGTCACAGCTTGGGGGTGACtctggtggtt
ctACTAATCTGTCAGATATTATTGAAAAGGAGACCGGTAAGCAACTGGTTATCCAGGAATCCATCCTCATGCTCCCAGAGGAGGTGGAAG
AAGTCATTGGGAACAAGCCGGAAAGCGATATACTCGTGCACACCGCCTACGACGAGAGCACCGACGAGAATGTCATGCTTCTGACTAGCG
ACGCCCCTGAATACAAGCCTTGGGCTCTGGTCATACAGGATAGCAACGGTGAGAACAAGATTAAGATGCTCtctggtggttctCCCAAGAAGA
AGAGGAAAGTCgggcccGAGGGCAGAGGAAGTCTGCTAACATGCGGTGACGTCGAGGAGAATCCTGGCCCAACTAATCTGTCAGATATTAT
TGAAAAGGAGACCGGTAAGCAACTGGTTATCCAGGAATCCATCCTCATGCTCCCAGAGGAGGTGGAAGAAGTCATTGGGAACAAGCCGG
AAAGCGATATACTCGTGCACACCGCCTACGACGAGAGCACCGACGAGAATGTCATGCTTCTGACTAGCGACGCCCCTGAATACAAGCCTT
GGGCTCTGGTCATACAGGATAGCAACGGTGAGAACAAGATTAAGATGCTCCCCAAGAAGAAGAGGAAAGTCGCTACTAACTTCAGCCTG
CTGAAGCAGGCTGGAGACGTGGAGGAGAACCCTGGACCTACCAACCTGTCCGATATCATTGAGAAAGAGACCGGCAAACAGCTGGTGATC
CAGGAGAGCATCCTGATGCTGCCCGAAGAGGTGGAGGAAGTGATCGGCAACAAGCCCGAGTCCGACATCCTGGTGCACACAGCCTATGAT
GAATCCACCGACGAGAACGTGATGCTGCTGACCTCCGATGCTCCCGAGTATAAACCCTGGGCACTGGTGATCCAGGACTCTAATGGAGAG
AACAAGATCAAGATGCTGCCCAAGAAGAAGAGGAAAGTCGAAGGACGGGGCTCCCTCCTGACCTGTGGCGATGTGGAAGAGAACCCCGG
CCCCACAAACCTCAGTGACATTATCGAGAAGGAAACAGGAAAACAGCTCGTCATTCAAGAATCTATTCTTATGTTGCCTGAGGAAGTCGA
AGAGGTTATTGGCAATAAACCTGAATCTGATATTCTTGTCCATACCGCTTACGATGAGTCCACAGATGAAAATGTTATGCTGCTCACATC
TGACGCACCAGAGTACAAACCATGGGCGCTCGTTATTCAAGATTCCAACGGCGAAAACAAAATCAAAATGCTTtctggtggttctCCTAAAAA
AAAAAGAAAGGTG**TGA**

Sequences for APOBEC1, nCas9, UGI , NLS, Flag and 2A are labeled in red, brown, blue, black, purple and cyan, respectively. The initiation and stop codons are highlighted in bold and sequences for linker peptides are indicated in lower case.
